# Stomatin-like protein 2 is overexpressed in epithelial ovarian cancer and predicts poor patient survival

**DOI:** 10.1186/s12885-015-1723-x

**Published:** 2015-10-20

**Authors:** Fei Sun, Wen Ding, Jie-Hua He, Xiao-Jing Wang, Ze-Biao Ma, Yan-Fang Li

**Affiliations:** 1 Department of Gynecologic Oncology, Sun Yat-sen University Cancer Center; State Key Laboratory of Oncology in South China; Collaborative Innovation Center of Cancer Medicine, 651 Dongfeng Road East, Guangzhou, 510060 P.R. China; 2 Department of Pathology, Sun Yat-sen University Cancer Center; State Key Laboratory of Oncology in South China; Collaborative Innovation Center of Cancer Medicine, 651 Dongfeng Road East, Guangzhou, 510060 P.R. China; 3 Department of Obstetrics and Gynecology, Guangzhou Women and Children’s Medical Center, 9 JinSui Road, 510623 Guangzhou, P.R. China; 4 Present address: Department of Obstetrics and Gynecology, Nanfang Hospital, Southern Medical University, Guangdong, 510515 P.R. China

**Keywords:** SLP-2, Epithelial ovarian cancer, Prognosis, Biomarker

## Abstract

**Background:**

Stomatin-like protein 2 (SLP-2, also known as STOML2) is a stomatin homologue of uncertain function. SLP-2 overexpression has been suggested to be associated with cancer progression, resulting in adverse clinical outcomes in patients. Our study aim to investigate SLP-2 expression in epithelial ovarian cancer cells and its correlation with patient survival.

**Methods:**

SLP-2 mRNA and protein expression levels were analysed in five epithelial ovarian cancer cell lines and normal ovarian epithelial cells using real-time PCR and western blotting analysis. SLP-2 expression was investigated in eight matched-pair samples of epithelial ovarian cancer and adjacent noncancerous tissues from the same patients. Using immunohistochemistry, we examined the protein expression of paraffin-embedded specimens from 140 patients with epithelial ovarian cancer, 20 cases with borderline ovarian tumours, 20 cases with benign ovarian tumours, and 20 cases with normal ovarian tissues. Statistical analyses were applied to evaluate the clinicopathological significance of SLP-2 expression.

**Results:**

SLP-2 mRNA and protein expression levels were significantly up-regulated in epithelial ovarian cancer cell lines and cancer tissues compared with normal ovarian epithelial cells and adjacent noncancerous ovarian tissues. Immunohistochemistry analysis revealed that the relative overexpression of SLP-2 was detected in 73.6 % (103/140) of the epithelial ovarian cancer specimens, 45.0 % (9/20) of the borderline ovarian specimens, 30.0 % (6/20) of the benign ovarian specimens and none of the normal ovarian specimens. SLP-2 protein expression in epithelial ovarian cancer was significantly correlated with the tumour stage (*P* < 0.001). Epithelial ovarian cancer patients with higher SLP-2 protein expression levels had shorter progress free survival and overall survival times compared to patients with lower SLP-2 protein expression levels. Multivariate analyses showed that SLP-2 expression levels were an independent prognostic factor for survival in epithelial ovarian cancer patients.

**Conclusions:**

SLP-2 mRNA and proteins were overexpressed in epithelial ovarian cancer tissues. SLP-2 protein overexpression was associated with advanced stage disease. Patients with higher SLP-2 protein expression had shorter progress free survival and poor overall survival times. Thus, SLP-2 protein expression was an independent prognostic factor for patients with epithelial ovarian cancer.

## Background

Epithelial ovarian cancer accounts for 80 %−90 % of ovarian cancers and is the leading cause of death in patients with gynaecologic malignancies [1] . The absence of specific symptoms and lack of reliable early diagnostic methods has resulted in the diagnosis of 70 % of patients at an advanced stage [2]. Despite progress in the development of new therapeutic methods, the 5-year survival rate of epithelial ovarian cancer patients has remained at approximately 30 % [3]. Epithelial ovarian cancer is thought to arise from an accumulation of genetic changes in a manner similar to other cancers [4]. Therefore, understanding the molecular mechanisms of the early events of epithelial ovarian cancer and searching for novel biomarkers involved in the progression of epithelial ovarian cancer is of great value for the identification of early-stage patients, providing new therapeutic targets, and improving patient survival.

Stomatin-like protein 2 (SLP-2, also known as STOML2) is a major protein on the mitochondrial inner membrane and a member of the stomatin superfamily. The relatively conserved 31-kDa protein has been shown to interact with prohibitin-1 and−2 [5, 6]. However, human SLP-2 has very low overall homology compared with other stomatins because SLP-2 lacks the characteristic amino-terminal transmembrane domain. SLP-2 may play an important role in organizing sphingolipid and cholesterol-rich lipid rafts, regulating ion channel conductance, and linking other integral membrane proteins to the peripheral cytoskeleton [5]. Previous studies revealed that human SLP-2 is a novel cancer-related gene of unknown function. The SLP-2 protein was first found to be overexpressed in human oesophageal cancer. Transecting antisense SLP-2 into the oesophageal squamous cell carcinoma cell line TE12 reduced cell growth and adhesion. These results suggested that SLP-2 was a potential oncogene [7, 8]. Further studies showed that the SLP-2 protein was overexpressed in many human cancer tissues, including gastric cancer [9], endometrial adenocarcinoma [10], and breast cancer [11]. SLP-2 up-regulation is correlated with the transformation of normal cells into tumour cells by an unknown mechanism. Thus, SLP-2 expression levels or copy number status may serve as a useful prognostic factor for cancer patients [10]. However, the expression status of SLP-2 and its clinical significance in epithelial ovarian cancer remain unclear. We investigated the protein and mRNA expression levels of SLP-2 in ovarian cancer tissues using immunohistochemistry, western blotting, and RT-PCR to analyse the potential clinical significance of SLP-2 expression.

## Methods

### Cell culture

OVCAR3 and Anglne cells were purchased from the China Center for Type Culture Collection (CCTCC, Wuhan, China). OVCAR3 cells were grown in RPMI 1640 supplemented with 10 % FBS, and Anglne cells were grown in Eagle’s minimal essential medium (Eagle’s MEM) supplemented with 10 % FBS. SKOV3 and HO8910 cells were purchased from the Shanghai Cell Bank of the Chinese Academy of Science (Shanghai, China). SKOV-3 cells were grown in McCoy’s 5A medium supplemented with 10 % FBS and HO8910 cells were grown in RPMI 1640 medium (HyClone, Logan, UT, USA) supplemented with 10 % FBS. A2780 cells (Nanjing KeyGen Biotech, Nanjing, China) were cultured in high glucose DMEM supplemented with 10 % FBS. Primary normal ovarian surface epithelial (NOSE) cells were established according to the method described in previous reports [12].

### Tissue samples and patient information

For real-time PCR and western blotting analysis, eight matched pairs of fresh tumour tissue specimens and adjacent noncancerous tissue samples were obtained from patients with epithelial ovarian cancer immediately after surgery and immersed at−80 °C until use. The percentages of tumour purity in these tissues and adjacent sections used for RNA and protein analyses were established by routine histopathological analyses. For immunohistochemistry, a total of 140 cancer tissue samples were collected from patients with epithelial ovarian cancer, 20 from patients with borderline ovarian tumours, and 20 from patients with benign ovarian tumours. Additionally, 20 normal ovarian epithelial tissues were collected from patients with benign uterine tumours who needed a hysterectomy and oophorectomy. All patients received surgery. Most patients (except those who had stage IA and grade 1 tumors) had post-operation adjuvant chemotherapy with platinum-based regimen. The patient list was obtained from the database of Sun Yat-sen University Cancer Center. Patient hospital records were reviewed to obtain demographic data, including age, serum levels of CA125, diagnosis, volume of ascites, surgical procedures, tumour stage, pathological reports, post-operation chemotherapy, and results of follow-up. All patient tissue samples were histologically confirmed to be epithelial ovarian cancers; these patients received treatment at the Sun Yat-sen University Cancer Center between January 1, 2003, and December 31, 2008. None of the patients had received prior radiotherapy or chemotherapy.

Eight matched pairs of fresh tumour tissue specimens and adjacent noncancerous tissue samples were collected from eight patients with serous epithelial ovarian cancer. Of these eight patients, three had stage I disease, two had stage II, and three had stage III; additionally, one patient had a grade 1–2 tumour, three had grade 2 tumours, two had grade 3 tumours, and two had grade 2–3 tumours. Adjacent noncancerous tissue samples were collected from either the noncancerous stroma of the same ovary (Patients 1–5, who had stage I or II tumours) (Fig. 2) or from the normal stroma of the contra-lateral ovary (Patients 6–8, who had stage III tumours) (Fig. 2). Of the 20 patients with borderline tumours, ten had serous tumours, seven had mucinous tumours, two had mixed tumours, and one had another type. Of the 20 patients with benign tumours, 16 had serous and four had mucinous tumours. All patients with ovarian cancer received surgery. Most patients (except those who had stage IA and grade 1 tumours) had post-operation adjuvant chemotherapy with a platinum-based regimen. Clinical follow-up data were available until December 31, 2013. The clinical information on the 140 patients with ovarian cancer whose tumour tissues were used for immunohistochemistry is summarized in Table 1. Patient’s consent was waived for this study since every patient at our institute have signed an informed consent on admission time for future possible use of the tumour sample for scientific research. Our study was approved by Sun Yat-sen University Cancer Center IRB (Approval No: B2014-2-26).

**Table 1:** Clinicopathological characteristics of patients with epithelial ovarian cancer and their correlations with SLP-2 expression

Characteristics	Number of cases (%)	SLP-2 expression (%)		*P* value
		Low or no expression	High expression	
Age (years)				0.433
<45	49 (35)	11 (22.4)	38 (77.6)	
≥45	91 (65)	26 (29.7)	65 (70.3)	
CA125 level (before surgery)				0.067
<500U/ml	67 (47.9)	23 (34.3)	44 (65.7)	
≥500U/ml	73 (52.1)	15 (20.5)	58 (79.5)	
Tumour size				0.501
<10 cm	58 (41.4)	14 (24.1)	44 (75.8)	
≥10 cm	82 (58.6)	24 (29.3)	58 (70.7)	
The volume of ascites				0.014
<1000 ml	100 (71.4)	33 (33.0)	67 (67.0)	
≥1000 ml	40 (28.6)	5 (12.5)	35 (87.5)	
Peritoneal cytology				0.001
Positive	68 (48.6)	10 (14.7)	58 (85.3)	
Negative	72 (51.4)	28 (38.9)	44 (61.1)	
Pathological type				0.064
Serous	80 (57.1)	20 (25.0)	60 (75.0)	
Mucinous	14 (10.0)	8 (57.1)	6 (42.9)	
Poorly differentiated	36 (25.7)	8 (22.2)	28 (77.8)	
Others^a^	10 (7.1)	2 (20.0)	8 (80.0)	
Grade of differentiation				0.072
G1	59 (42.1)	11 (18.6)	48 (81.4)	
G2	46 (32.9)	13 (28.3)	33 (71.7)	
G3	18 (12.9)	9 (50.0)	9 (50.0)	
Unknown	17 (12.1)	5 (29.4)	12 (70.6)	
FIGO stage				0.001
I	30 (21.4)	17 (56.7)	13 (43.3)	
II+ III + IV	110 (78.6)	21 (19.1)	89 (88.9)	
Lymph node metastasis				0.688
Positive	21 (15.0)	7 (33.3)	14 (66.7)	
Negative	23 (16.4)	5 (21.7)	18 (78.3)	
Not do RPLND^b^	96 (68.6)	26 (27.1)	70 (72.9)	
Cytoreductive surgery^c^				0.185
Optimal	107 (76.4)	32 (29.9)	75 (70.1)	
Suboptimal	33 (23.6)	6 (18.2)	27 (81.8)	

^a^Endometrioid adenocarcinoma, two cases; clear cell carcinoma, three cases; mixed epithelial carcinoma, five cases

^b^RPLND, retroperitoneal lymph node dissection, including unilateral or bilateral pelvic lymphadenectomy and /or paraortic lymphadenectomy

^c^Cytoreductive surgery: Optimal, the diameter of the largest residual lesions was < 2 cm;Suboptimal, the diameter of the largest residual lesions was ≥ 2 cm

### Real-time PCR (RT-PCR)

Total RNA samples were extracted from cultured cells and primary tumour tissues using the TRIzol reagent (Invitrogen, Carlsbad, CA, USA) in accordance with the manufacturer’s instructions and treated with RNase-free DNase. cDNA was synthesized from 2 μg of RNA from each sample using an iScript™ cDNA Synthesis Kit (BioRad Laboratories, Hercules, CA, USA). The RT-PCR cycling conditions incorporated an initial denaturation at 94 °C for 5 min, followed by 30 denaturation cycles at 94 °C for 30 s, primer annealing at 55 °C for 30 s, primer extension phase at 72 °C 50 s, and a final extension step at 72 °C for 7 min. The primers for SLP-2 and glyceraldehyde-3-phosphate dehydrogenase (GAPDH) were designed using Primer Express v 2.0 software (Applied Biosystems). The sequences of the primers were as follows: forward primer 5’-GTGACTCTCGACAATGTAAC-3’ and reverse primer 5’-TGATCTCATAACGGAGGCAG -3’. SLP-2 expression data were normalized to GAPDH, and all experiments were performed in triplicate.

### Western blotting

The cells were washed twice with ice-cold phosphate-buffered saline (PBS) and lysed on ice in radio immunoprecipitation assay (RIPA) buffer (Cell Signaling Technology, Danvers, MA) containing complete protease inhibitor cocktail (Roche Applied Science, Mannheim, Germany). Fresh tissue samples were ground to powder in liquid nitrogen and lysed with SDS-PAGE sample buffer. All protein samples (20 μg) were separated on 12 % sodium dodecyl sulfate–polyacrylamide gels, transferred to polyvinylidene fluoride (PVDF) membranes (Immobilon P, Millipore, Bedford, MA) and blocked with 5 % skimmed milk in Tris-buffered saline supplemented with 0.1 % Tween 20 (TBST) for 1 h at room temperature. After blocking, the membranes were incubated with anti-SLP-2 antibodies (1:1000, Proteintech, Chicago, IL, USA) at 4 °C overnight. Then, the membranes were rinsed with TBST and incubated with an anti-rabbit IgG antibody (Santa Cruz Biotechnology, Santa Cruz, USA) conjugated to horseradish peroxide for 15 min. The expression of SLP-2 was detected with the enhanced chemiluminescence (ECL) prime western blotting detection reagent (Amersham Bioscience, Switzerland) according to the manufacturer’s instructions. An anti-ß-actin antibody (Sigma, St. Louis, MO) were used as a loading control.

### Immunohistochemistry

Immunohistochemical analysis was used to study SLP-2 protein expression in 140 epithelial ovarian cancer samples, 20 borderline ovarian tumour samples, 20 benign ovarian tumour samples and 20 normal ovarian epithelial tissues. Paraffin-embedded specimens were cut into 4-μm-thick sections, de-waxed with xylene and rehydrated. For antigenic retrieval, the sections were submerged into EDTA antigenic retrieval buffer and microwaved, and then treated with 3 % hydrogen peroxide in methanol to quench endogenous peroxidase activity. Subsequently, the sections were incubated with 1 % bovine serum albumin to block nonspecific binding, and then incubated with an anti-SLP-2 rabbit polyclonal antibody (1:1000, Proteintech, Chicago, IL, USA) overnight at 4 °C. Normal goat serum was used as the negative control. After washing, the sections were incubated with a biotinylated anti-rabbit secondary antibody (Abcam, Cambridge, MA), and then further incubated with a streptavidin-horseradish peroxidase complex (Abcam, Cambridge, MA). Finally, the tissue sections were immersed in 3.30-diaminobenzidine, counterstained with 10 % Mayer’s hematoxylin, dehydrated and mounted in crystal mount medium.

SLP-2 staining was scored by two independent pathologists. The scores were averaged based on both the intensity of staining and the proportion of positively stained tumour cells. The proportion of tumour cells was scored as follows: 0 (<5 % positive tumour cells), 1 (6–25 % positive tumour cells), 2 (26–50 % positive tumour cells), 3 (51–75 % positive tumour cells), and 4 (>75 % positive tumour cells). The intensity of staining was graded as follows: 0 (no staining); 1 (weak staining ~ light yellow), 2 (moderate staining ~ yellow brown), and 3 (strong staining ~ brown). The staining index for SLP-2 expression in epithelial ovarian cancer was calculated by multiplying the two scores of the proportion of positive cells and the intensity of staining. Cut-off values for SLP-2 were based on the median of all products. An optimal cut-off value was identified as follows: a score ≥ 6 was used to define tumours with high SLP-2 expression and a score ≤ 4 indicated low SLP-2 expression.

### Statistical analyses

All statistical analyses were conducted using the SPSS software package (IBM, standard version 16.0). The relationship between the expression of SLP-2 and clinicopathological characteristics was analysed by Pearson’s *χ*2 and Fisher’s exact tests. Overall survival (OS) was defined as the time from surgery to death or to the last follow-up. Progression-free survival (PFS) was defined as the length of time after treatment to the onset of recurrence or progression (diagnosed by imaging or clinical assessment). Kaplan–Meier curves were plotted to assess the effects of SLP-2 expression levels on PFS and OS, and survival curves were compared using a log-rank test. Multivariate Cox regression analysis was performed for all clinicopathological variables that were found to be significant by univariate analysis. In all tests, a two-sided *P*-value of less than 0.05 was considered to be statistically significant.

## Results

### The SLP-2 mRNA and protein were overexpressed in epithelial ovarian cancer cell lines

**Fig. 1:**
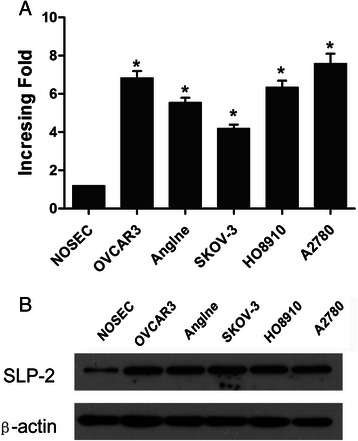
Overexpression of SLP-2 mRNA and protein in epithelial ovarian cancer cell lines. SLP-2 mRNA and protein expression in epithelial ovarian cancer cell lines (OVCAR3, Anglne, SKOV-3, HO8910, and A2780) and NOSE cells were examined by teal-time PCR (**a**) and western blotting (**b**). Expression levels were normalized against GAPDH and β-actin, respectively. Error bars represent standard deviation of the mean (SD) calculated from three parallel experiments. *P < 0.05

We used real-time RT-PCR and western blotting to investigate the mRNA and protein expression levels of SLP-2 in five epithelial ovarian cancer cell lines (OVCAR3, Anglne, SKOV-3, HO8910 and A2780) and normal ovarian surface epithelial (NOSE) cells. The mRNA expression of SLP-2 was at least 4-fold higher in epithelial ovarian cancer cell lines than in the NOSE cells (Fig. 1a). Moreover, the SLP-2 protein was highly expressed in the epithelial ovarian cancer cell lines and only weakly expressed in the NOSE cells (Fig. 1b).

### The SLP-2 mRNA and protein were overexpressed in epithelial ovarian cancer tissues

**Fig. 2:**
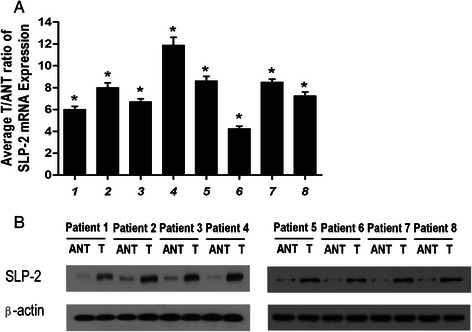
Overexpression of SLP-2 mRNA and protein in epithelial ovarian cancer tissues. **a** Average T/ANT ratios of SLP-2 mRNA expression in paired epithelial ovarian cancer tissues (T) and adjacent noncancerous tissues (ANT) were quantified by qPCR and normalized against GAPDH. Error bars represent the standard deviation of the mean (SD) calculated from three parallel experiments. *P < 0.05. **b** Representative images of western blotting analyses of SLP-2 protein expression in eight matched pairs of epithelial ovarian cancer tissues (T) and adjacent noncancerous tissues (ANT). β-actin was used as the loading control

**Fig. 3:**
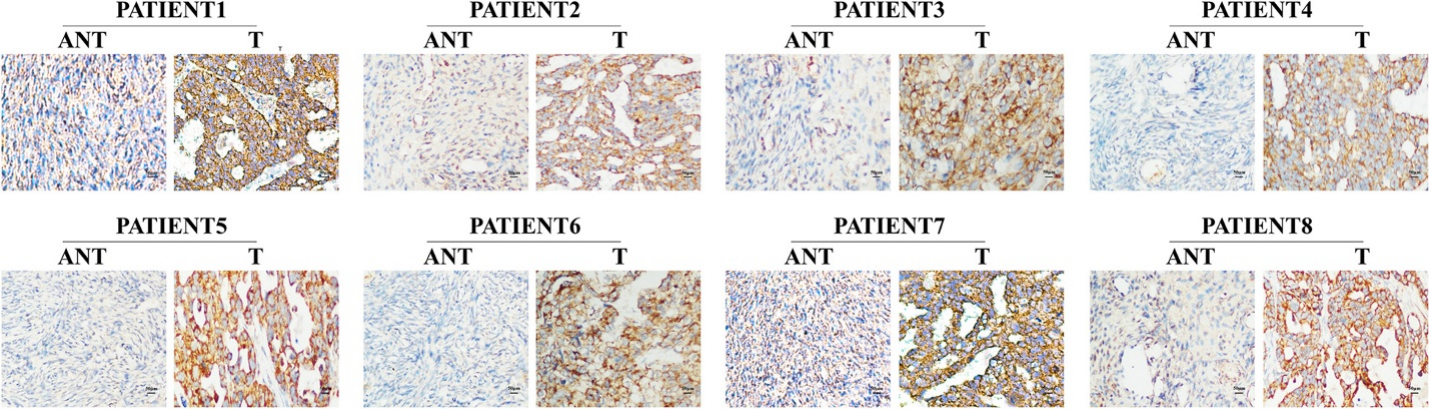
Immunohistochemical assay of SLP-2 protein expression in eight pairs of matched epithelial ovarian cancer tissues

To investigate the SLP-2 mRNA and protein expression levels in human epithelial ovarian cancer tissues, we used real-time RT-PCR and western blotting to analyse eight matched pairs of epithelial ovarian cancer specimens (T) and adjacent noncancerous tissue samples (ANT). SLP-2 mRNA was expressed at higher levels in all epithelial ovarian cancer tissues compared to adjacent noncancerous tissues, with the differential expression levels ranging from 4.4- to 11.8-fold (Fig. 2a). Additionally, the SLP-2 protein was also up-regulated in epithelial ovarian cancer tissues compared with the matched noncancerous tissues (Fig. 2b, Fig. 3). SLP-2 was mainly located in the cell membrane and cytoplasm.

### SLP-2 protein expression was higher in epithelial ovarian cancers than in benign and borderline ovarian tumours

**Fig. 4:**
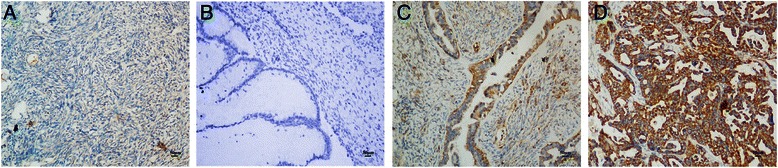
SLP-2 protein expression in ovarian epithelial cancer tissues and the control group. **a** normal ovarian epithelial tissues, **b** benign epithelial ovarian tumour, **c** borderline epithelial ovarian tumour, **d** epithelial ovarian cancer

**Fig. 5:**
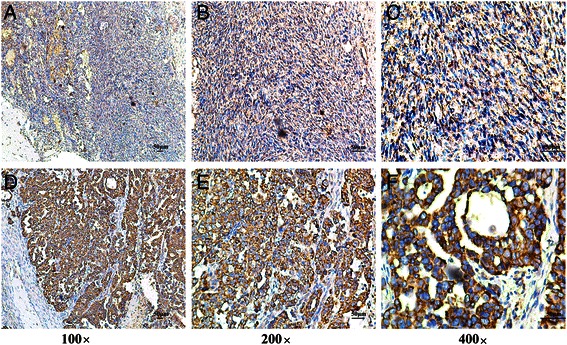
SLP-2 protein expression in epithelial ovarian cancer sections. Representative immunohistochemical images of epithelial ovarian cancer tissue specimens indicating strong SLP-2 staining (**d**, **e** and **f**) and weak or negative detectable SLP-2 staining in normal ovarian epithelial tissues (**a**, **b** and **c**). Magnification × 100 (**a** and **d**), × 200 (**b** and **e**) or × 400 (**c** and **f**)

To compare the difference in SLP-2 expression between epithelial ovarian cancer and benign and borderline ovarian tumours, we examined paraffin-embedded archived samples from 140 cases of epithelial ovarian cancer tissues, 20 borderline ovarian tumour tissues and 20 benign ovarian tumour tissues; additionally, 20 normal ovarian epithelial tissues were included as the control group. SLP-2 protein expression was analysed by immunohistochemical staining. High SLP-2 protein expression was detected in 72.9 % (102/140) of epithelial ovarian cancer samples, in 45.0 % (9/20) of borderline ovarian tumour tissues, in 30.0 % (6/20) of benign ovarian tumour tissues, and in none (0/20) of the normal ovarian epithelial tissues (Table 2, Figs. 4 and 5). The 6 cases of benign tumours with SLP-2 overexpression included 5 serous and 1 mucinous type; The 9 cases of borderline ovarian tumour with SLP-2 overexpression included 6 serous tumour, 2 mucinous tumour, and 1 mixed tumour. Thus, SLP-2 protein expression in epithelial ovarian cancer samples was higher than benign ovarian tumours and borderline ovarian tumours (both *P* < 0.001) (Table 2).

**Table 2:** SLP-2 protein expression in the epithelial ovarian cancer group and the control groups

Group	Number of cases	SLP-2 expression (%)		*χ* ^2^	*P* value
		Low or no expression	High expression		
1 Epithelial ovarian cancer	140	38 (27.1)	102 (72.9)	6.803	<0.001^a c d^
2 Borderline ovarian tumour	20	11 (55)	9 (45)	15.300	<0.001^b^
3 Benign ovarian tumour	20	14 (70)	6 (30)	12.764	<0.001
4 Normal ovarian epithelial tissues	20	20 (100)	0 (0)	41.303	<0.001

^a^Comparison between group 1 and group 2

^b^Comparison between group 2 and group 3

^c^Comparison between group 1 and group 3

^d^Comparison between group 1 and group 4

### SLP-2 overexpression was associated with epithelial ovarian cancer clinical features

Out of the 140 patients with epithelial ovarian cancer, 30 patients had stage I tumours, 23 had stage II tumours, 77 had stage III tumours, and 10 had stage IV tumours. The median age was 46 years (range, 15 ~ 76 years). All 140 patients received initial treatment, including surgery and post-operation chemotherapy.

Statistical analysis showed a significant correlation between SLP-2 protein expression and the clinicopathological characteristics of epithelial ovarian cancer, including tumour stage (*P* < 0.001), peritoneal cytology (*P* < 0.001), and the ascites volume (*P* = 0.014). In contrast, SLP-2 expression did not correlate with age, CA125 levels, tumour sizes and other clinicopathological characteristics (Table 1). Logistic multivariate analysis showed that the SLP-2 protein overexpression level was associated with the tumour stage (*P* = 0.049), but was not associated with peritoneal cytology and the ascites volume (*P* > 0.05). Patients with late stage disease had higher SLP-2 protein expression levels compared to patients with early stage tumours (Table 1).

### Relationship between SLP-2 expression and patient survival

We performed a Kaplan-Meier analysis to investigate the relationship between SLP-2 expression and the survival of patients with epithelial ovarian cancer. At the last clinical follow-up, 86 out of 140 patients were alive and 54 were dead, and the median follow-up time was 52 months (range, 1 ~ 121 months). The median progress free survival (PFS) and overall survival (OS) for all patients was 33 and 52 months, respectively.

**Fig. 6:**
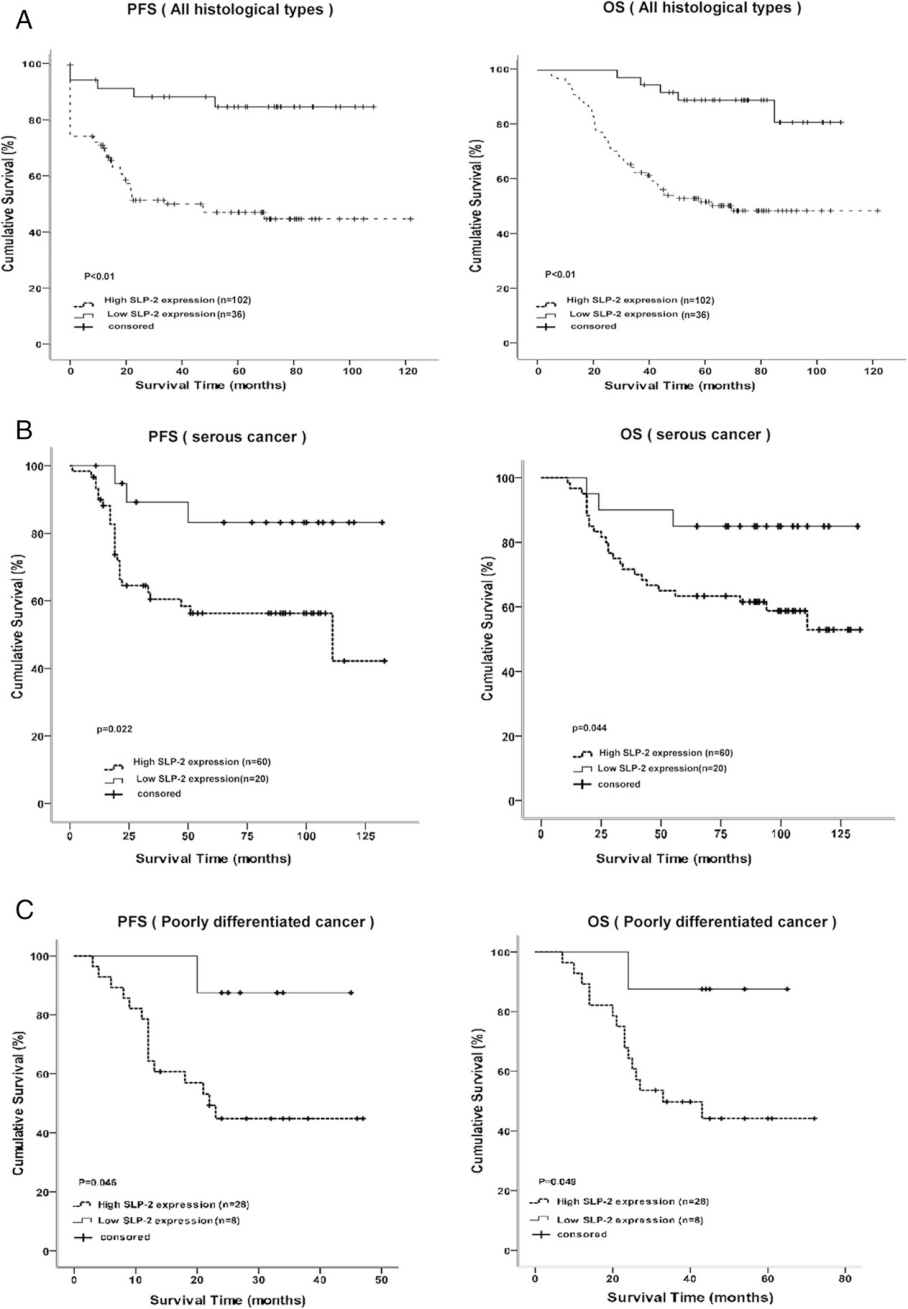
The level of SLP-2 protein expression affects progression free survival and overall survival. **a** Kaplan–Meier curves with univariate analysis (log-rank) for epithelial ovarian cancer patients with high SLP-2 expression (n = 102) versus low or no SLP-2 expression (n = 38) for progression free survival and overall survival for all histological types. **b** Kaplan–Meier curves with univariate analysis (log-rank) for epithelial ovarian cancer patients with high SLP-2 expression (n = 60) versus low or no SLP-2 expression (n = 20) for progression free survival and overall survival for serous types. **c** Kaplan–Meier curves with univariate analysis (log-rank) for epithelial ovarian cancer patients with high SLP-2 expression (*n* = 28) versus low or no SLP-2 expression (*n* = 8) for progression free survival and overall survival for poorly differentiated types

The median PFS of patients with high and low/no SLP-2 expression was 19 months (range, 1 ~ 121 months) and 61 months (range, 1 ~ 108 months), respectively (Log-rank test *χ*2 = 14.79,*P* < 0.001). The median OS of patients with high and low/no SLP-2 expression was 46 months (range, 4 ~ 121 months) and 74 months (range, 1 ~ 108 months), respectively (Log-rank test *χ*2 = 15.39,*P* < 0.001). These results suggested a clear negative correlation between the level of SLP-2 protein expression and both the PFS and OS of patients with epithelial ovarian cancer (both *P* < 0.01, Fig. 6a).

To determine whether SLP-2 protein expression could serve as an independent prognostic factor, we examined PFS and OS using the Cox proportional hazards model. We examined several potential prognosis-related factors, including age, tumour stage, peritoneal cytology, ascites volume, preoperative CA125 levels, tumour size, histological type, tumour cell differentiation, lymph node metastasis, and residual tumours. Univariate analysis revealed that the tumour stage, peritoneal cytology, ascites volume and SLP-2 overexpression were associated with PFS and OS. Further analysis with a multivariate COX model showed that only tumour stage (*P* = 0.04), optimal cytoreductive surgery (*P* = 0.003) and SLP-2 overexpression (*P* = 0.023) were independent prognostic factors for poor PFS. Similarly, Cox regression analysis revealed that tumour stage (*P* = 0.04), optimal cytoreductive surgery (*P* = 0.001), pathological type (*P* = 0.019) and SLP-2 overexpression (*P* = 0.009) were also independent prognostic factors for poor OS.

Next, we performed survival analysis in two subgroups (serous cancer and poorly differentiated) that possessed a larger sample size. Univariate analysis revealed that SLP-2 overexpression was associated with poor PFS (*P = 0.022*) and OS *(P = 0.044*) in the 80 patients with serous cancer (Fig. 6b), while Cox regression analysis showed that highertumour stage, positive peritoneal cytology, and SLP-2 overexpression were independent prognostic factors for both poor PFS (*P = 0.05*, *0.001, and 0.003, respectively*) and OS (*P = 0.004, 0.004, and 0.01, respectively*). In 36 patients with poorly differentiated cancer, univariate analysis revealed that SLP-2 overexpression was associated with poor PFS (*P = 0.046*) and OS (*P = 0.049*) (Fig. 6c); Cox regression analysis showed that SLP-2 overexpression was associated with OS (*P = 0.023*), but was not associated with PFS (*P = 0.058*). The other factors mentioned above were not associated with either PFS or OS (*P > 0.05*).

### Validation of the prognostic value of SLP-2 in ovarian cancer series from publicly available datasets

**Fig. 7:**
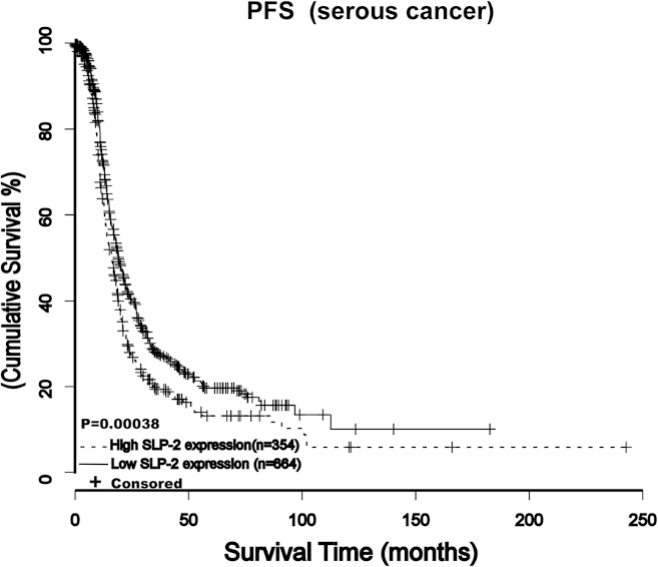
The prognostic value of SLP-2 in ovarian cancer series from publicly available datasets. Kaplan–Meier curves with univariate analysis (log-rank) for epithelial ovarian cancer patients with high SLP-2 expression (*n* = 354) versus low SLP-2 expression (*n* = 664) for progression free survival for serous types

We evaluated the prognostic value of SLP-2 in ovarian cancer using online Kaplan-Meier plotter (*http://kmplot.com/analysis/index.php?p=service&cancer=ovar*), which integrates gene expression and clinical data from 12 different data sets from 1648 patients [13]. We found that higher mean SLP-2 protein expression in 354 patients was associated with shorter PFS as compared with that in the 664 patients with lower SLP-2 protein expression with serous ovarian cancer (HR = 1.33, Logrank *P* = 0.00038, Fig. 7). These results further suggested that SLP-2 protein expression is associated with prognosis and higher SLP-2 protein expression predicts poorer patient’s survival

## Discussion

In this study, we showed that the SLP-2 mRNA and protein were overexpressed in epithelial ovarian cancer. SLP-2 protein overexpression was associated with late stage disease. The expression of the SLP protein was an independent prognostic factor in patients with epithelial ovarian cancer. To the best of our knowledge, this is the first study on SLP-2 expression in patients with ovarian cancer.

Studies have shown that SLP-2 is a potential oncogene. It was first found to be up-regulated in human oesophageal cancer cells [14, 15]. Knockdown of STOML2 reduced the growth rate of oesophageal cancer cells in vitro and in vivo and inhibited cell attachment [8]. SLP-2 was also found to be over-expressed in other cancers, including endometrial cancer, lung cancer, laryngeal cancer, and breast cancer [8, 11]. Over-expression of SLP-2 in cancer tissues was associated with decreased patient survival and was an independent prognostic factor for lung cancer [16], breast cancer [11], gastric cancer [9], and glioma [17].

The exact mechanism underlying SLP-2 involvement in tumourigenesis and development remains unclear. Wang Y et al. reported that SLP-2 may be involved in bioenergetics in the mitochondria. Mitochondrial membrane potential (MMP) is an important physiological parameter that reflects the mitochondria status. MMP alterations lead to changes in cellular ATP production, which supplies energy to maintain cell activity. Knock-down of SLP-2 by siRNA in oesophageal squamous cell carcinoma KYSE 150 cells reduced MMP, decreased the ATP level, and potently inhibited cell motility and proliferation [18]. Up-regulation of SLP-2 was effectively abrogated by ERK1/2 inhibitors, and the regulation of SLP-2 was proposed to be involved in the activation of the MAPK/ERK pathway [19]. Song L et al. showed that the invasive ability of glioma cells was reduced by knockdown of SLP-2 through inhibition of the NF-κB/MMP-9 pathway [17].

We demonstrated that the mRNA and protein levels of the SLP-2 gene were overexpressed in epithelial ovarian cancer cells. Based on our RT-PCR and western blotting results, SLP-2 mRNA and protein expression levels were higher in epithelial ovarian cancer cell lines than in NOSE cells (Figs. 1a and b). Additionally, the SLP-2 mRNA and protein were expressed at higher levels in fresh epithelial ovarian cancer tissues than in adjacent noncancerous tissues. Using immunohistochemical staining, we demonstrated that SLP-2 protein expression was higher in epithelial ovarian cancer cells than in benign and borderline ovarian tumours. All of these results suggested that the SLP-2 mRNA and protein were overexpressed in epithelial ovarian cancers.

Our study demonstrated that SLP-2 overexpression was associated with disease progression and poor survival outcomes for patients with epithelial ovarian cancer, and thus SLP-2 may be regarded as a potential prognostic factor. The standard treatment for epithelial ovarian cancer is surgery, followed by post-operation chemotherapy. Despite the improvement in surgical skills and emergence of new chemotherapeutic agents and methods [2], the overall survival of patients with epithelial ovarian cancer has remained poor, with a 5-year survival rate of approximately 30 % [3]. This is mainly because approximately 70 % of patients have late-stage disease at the time of diagnosis, and relapse occurs in approximately 80 ~ 90 % of the patients [1, 2]. Known prognostic factors that can predict recurrence and survival include the stage, size of the post-operative residual tumour, and lymph node metastasis. However, patients with these factors may have different prognoses, which suggests that other factors may also be present and affect patient prognosis (i.e., molecular biomarkers). Thus, it is important to search for new prognostic factors to enable better predictions of patient prognosis and assist with decisions about treatment options. SLP-2 may be such a prognostic factor. Using immunohistochemical staining, we showed that the SLP-2 protein overexpression level was associated with the tumour stage; patients with late stage disease had higher SLP-2 protein expression levels than those with early stage tumours. Further analysis showed that higher SLP-2 protein expression was significantly associated with shorter PFS time and poorer OS of patients with epithelial ovarian cancer. Multivariate analyses revealed that SLP-2 expression was an independent prognostic factor for patient survival. Stratified analysis in subgroups also showed that SLP-2 protein overexpression was an independent prognosis factor for patients with the most common type of epithelial ovarian cancer (serous cancer). These results suggest that SLP-2 is involved in the progression of epithelial ovarian cancer and that SLP-2 overexpression is predictive of poor patient survival.

The question as to why the overexpression of SLP-2 leads to poor patient prognosis remains. One possible reason may be that up-regulated SLP-2 renders cancer cells resistant to chemotherapy. Post-operation chemotherapy plays an important role in the treatment of ovarian cancer. Patients with tumours overexpressing SLP-2 may exhibit a poorer response to chemotherapy than patients with tumours expressing low levels of SLP-2. Although we did not have clinical data to support this hypothesis in our study, preclinical studies in the literature may provide us with some suggestions. Tondera D et al. [20] showed that SLP-2 is required for stress-induced mitochondrial hyperfusion (SIMH); SIMH confers cells with resistance to stressors such as chemotherapeutic agents. In Wang Y et al.’s study, SLP-2 depletion enhanced the sensitivity to the chemotherapeutic agent adriamycin in siRNA-transfected oesophageal squamous cell carcinoma cells YYYY. These results suggest that SLP-2 is chemotherapy-resistant related and also suggested that SLP-2 is a potential target for enhancing cancer chemotherapy.

## Conclusions

In this study, we showed that the SLP-2 mRNA and protein were overexpressed in epithelial ovarian cancer. SLP-2 protein overexpression was associated with advanced stage disease. Patients with higher SLP-2 protein expression levels had shorter PFS and poor OS. The expression of the SLP-2 protein was an independent prognostic factor for patients with epithelial ovarian cancer.

## Abbreviations

ANT: Adjacent noncancerous tissue
NOSE: Normal ovarian surface epithelial
OS: Overall survival
PFS: Progress free survival
PVDF: Polyvinylidene fluoride
SLP-2: Stomatin-like protein 2
TBST: Tris-buffered saline with 0.1 % Tween 20
